# Generating imperceptible adversarial examples via low-frequency aware transfer attacks on battery management systems

**DOI:** 10.1038/s41598-026-49020-1

**Published:** 2026-04-17

**Authors:** Kyeongseo Min, Yeseo Joo, Jiho Hong, Hoki Kim, Sangho Lee, Youngdoo Son

**Affiliations:** 1 Department of Industrial and Systems Engineering, Dongguk University-Seoul, Seoul, 04620 Republic of Korea; 2 Data Science Laboratory (DSLAB), Dongguk University-Seoul, Seoul, 04620 Republic of Korea; 3 Department of Industrial Security, Chung-Ang University, Seoul, 06974 Republic of Korea; 4 School of Industrial and Systems Engineering, Gyeongsang National University, Jinju, 52828 Republic of Korea

**Keywords:** Energy science and technology, Engineering, Mathematics and computing

## Abstract

Despite the success of deep learning models for the state-of-health estimation of lithium-ion batteries in battery management systems, their susceptibility to adversarial attacks raises concerns about security risks, leading to misdiagnosis and unnecessary maintenance. However, their practical robustness against adversarial perturbations remains largely underexplored, despite its importance for real-world deployment. Thus, we develop a novel adversarial attack to unveil potential risks associated with battery management systems. Our approach assesses model robustness in practical scenarios where model information is inaccessible by selectively exploiting frequency information that benefits the capture of unique characteristics for the state of health. In particular, we generate a *malicious* but *imperceptible* example by manipulating low-frequency components of a signal while preserving their relationships. Through a series of experiments, we demonstrate the effectiveness of our approach in uncovering and understanding potential risks associated with deep learning models in battery management systems.

## Introduction

Lithium-ion batteries (LIBs) have recently powered various applications, such as electric vehicles and energy storage stations, owing to their high energy density and lightweight structure^1,2^. However, their low stability often causes several safety issues, including thermal runaway; hence, battery management systems (BMSs) monitor the state of health (SOH) of LIBs to extend their cycle life and replace them on time^3,4^.

Based on the recent success of deep learning in various industrial fields^5–8^, several studies for SOH estimation have also attempted to utilize deep learning models to handle the high complexity of their internal chemical reactions effectively^9,10^. In particular, most of them have focused on estimating the SOH by utilizing voltage signals obtained from LIBs, which frequently encompass important information related to their current capacity and aging status^11–13^.

However, one of the potential drawbacks of deep learning models is the susceptibility to subtle noise, especially maliciously corrupted examples called adversarial examples^14,15^. The malicious but subtle perturbations against the voltage signals make deep learning models in BMSs inaccurately estimate their SOHs, causing critical safety accidents or enormous financial losses^16,17^. Despite this potential security risk, to our knowledge, there is a lack of research to evaluate the actual robustness of deep learning-based SOH estimation models against adversarial examples. Thus, it is necessary to investigate the practical danger of malicious examples from adversarial attacks on BMSs.

**Fig. 1:**
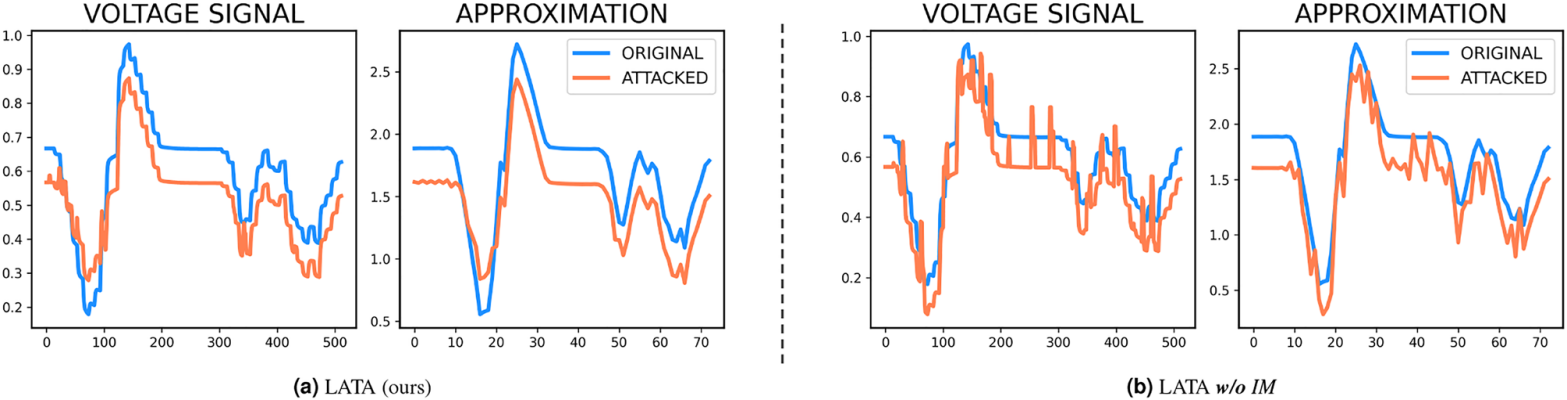
Empirical comparison of voltage signals and their approximation coefficients (**a**) ***with*** and (**b**) ***w/o*** *interaction map*.

To precisely estimate the practical robustness in BMSs by leveraging adversarial examples, two practical considerations should be addressed. First, in real-world deployments, it is challenging to fully access the deep learning model used for SOH estimation in BMSs. Accordingly, adversarial perturbations should be generated in a *black-box setting*, where the attacker can observe or manipulate only the input signals, while the internal architecture and parameters of the target model remain unknown^18–23^. This setting necessitates the design of transferable adversarial examples that generalize effectively across different models and architectures. Second, since most existing adversarial attacks, such as the fast gradient sign method (FGSM)^24^ and projected gradient descent (PGD)^25^, have been predominantly developed in the computer vision domain, it is difficult to consider *domain-specific knowledge*, which benefits evaluating the actual robustness of SOH estimation models^26^. In particular, voltage signals obtained from LIBs are generally created by the interference of several frequency components^27,28^, but the conventional methods attack the signals by solely focusing on their waveforms in the time domain while overlooking frequency information, which is insufficient to hinder the models. Moreover, as shown in Fig. 1b, even when considering frequency information, direct manipulation of frequency component values may damage their relationships. Such damages can cause adversarial examples to show different temporal variations from the original signals, which facilitates the detection of attacks. Thus, it is necessary to ensure imperceptibility when utilizing frequency information, which involves unique SOH characteristics, to accurately evaluate the SOH estimation models^26,29^.

Therefore, in this work, we design a novel adversarial attack method that generates *malicious* but *imperceptible* examples by effectively manipulating frequency information to evaluate the practical robustness of SOH estimation models under the black-box setting. Specifically, the proposed method generates adversarial examples by explicitly corrupting the low-frequency components of voltage signals, which indicate their underlying temporal patterns, such as trends and periodicities, crucial for SOH estimation^30,31^. Moreover, when manipulating the low-frequency components, we maintain their structural information by exploiting interaction maps derived from the outer product of the components to ensure the imperceptibility of the adversarial examples. As a result, our method can effectively attack deployed SOH estimation models in realistic black-box settings, requiring access only to input voltage signals and no knowledge of the target model architecture or parameters.

To demonstrate the superiority of our approach over the existing attack methods, we conducted a series of experiments on the LG18650HG2 LIB dataset^32^. Consequently, we revealed the potential risk of BMSs by validating that our approach generated malicious but imperceptible examples, as shown in Fig. 1a, achieving overwhelming performance degradation on various deep learning models compared to other existing attack methods.

The main contributions are summarized as follows:

- We propose a novel adversarial attack that generates transferable adversarial examples by selectively manipulating frequency components of voltage signals along with their waveforms, assessing the practical robustness of deep learning-based SOH estimation models in BMSs.
- To obtain *malicious* but *imperceptible* examples that obstruct the detection of attacks, we consider relationships between the low-frequency components by exploiting their outer product rather than directly manipulating them.
- Our approach remarkably reduces SOH estimation performance compared to existing methods under the black-box setting, uncovering potential risks of deep learning models in real-world BMSs.

The rest of this paper is structured as follows. We briefly review the SOH estimation and adversarial attacks against them in "Related work". In "Proposed method" describes the motivation behind our approach and details the proposed method. In "Experiments" , we present experimental settings and results, demonstrating the effectiveness of our method. Finally, in "Conclusion" summarizes our findings and concludes the paper.

## Related work

### State of health estimation

SOH estimation is one of the essential tasks in BMSs for the optimal management of LIBs^3,33^. Traditionally, experiment-based methods^34,35^, involving impedance measurement and cycle number counting, and model-based methods^36,37^, utilizing the electrochemical knowledge of LIBs to establish mathematical models describing battery degradation, have been suggested for SOH estimation. However, these methods often struggle to estimate SOH accurately due to the sophisticated and irreversible physical and chemical reactions occurring in LIBs^38^. Thus, recent studies have increasingly adopted deep learning-based approaches for SOH estimation, as they are capable of effectively modeling the highly complex degradation mechanism of LIBs^9,39,40^. For example, physics-guided long short-term memory (LSTM)^41^ and knowledge distillation-based framework^42^ have been proposed and deployed in practical BMS environments. In particular, many studies estimate SOH based on voltage signals collected from LIBs because these signals can provide rich information related to battery capacity and aging behavior that is crucial for SOH estimation^43^.

SOH is generally defined as the maximum charge that can be released after a LIB has been fully charged, as follows^44^:

1 $$\begin{aligned} SOH_t = Q_{t}/Q_{0}\times 100\%, \end{aligned}$$

where $Q_{t}$ is the current maximum charge of the LIB, and $Q_{0}$ is its initial rated capacity. In BMSs, the SOHs of LIBs are categorized into multiple stages to diagnose the life stage of LIBs and make decisions for their inspection or replacement^45,46^. For example, the SOH above 90% indicates a normal stage, whereas that under 90% is a degradation stage requiring some countermeasures. That is, we can treat the SOH estimation as a classification task in machine learning, which is referred to as *SOH diagnosis* in this paper. Let $\boldsymbol{x}$ be a voltage signal collected from the BMS of a LIB, and *y* be the corresponding life stage (or class) related to SOH. We classify the voltage signal $\boldsymbol{x}$ into the correct stage to identify the current degradation level of the LIB. The objective of this process is formulated by

2 $$\begin{aligned} \underset{f}{\min }~\mathscr {L}(f(\boldsymbol{x}), y), \end{aligned}$$

where *f* is a classifier that determines whether the voltage signal $\boldsymbol{x}$ corresponds to the stage *y*, and $\mathscr {L}$ is a certain loss function for the SOH diagnosis, such as cross-entropy.

### Adversarial attack

Although deep learning has significantly improved the SOH estimation and diagnosis performance for LIBs, its potential drawback is the vulnerability to adversarial attacks, causing security risks for BMSs^11,16^. The adversarial attack aims to learn malicious perturbation $\boldsymbol{\delta }$ to deceive deep learning models. Specifically, given a target model of the BMS, $f_\mathscr {T}$, the adversarial attack optimizes the following objective:

3 $$\begin{aligned} \max _{\Vert \boldsymbol{\delta }\Vert \le \varepsilon } \mathscr {L}(f_\mathscr {T}(\boldsymbol{x}+\boldsymbol{\delta }), y), \end{aligned}$$

where $\varepsilon$ is the maximum perturbation size. The resulting $\boldsymbol{\delta }$ deceives the given model $f_\mathscr {T}$ to satisfy $f_\mathscr {T}(\tilde{\boldsymbol{x}}) \ne f_\mathscr {T}(\boldsymbol{x})$ for the adversarial example $\tilde{\boldsymbol{x}}=\boldsymbol{x}+\boldsymbol{\delta }$.

In Goodfellow et al.^24^, they learned the perturbation $\boldsymbol{\delta }$ by proposing FGSM with the gradient ascent approach as follows:

4 $$\begin{aligned} \boldsymbol{\delta }= \varepsilon \cdot \texttt {sign}(\nabla _{\boldsymbol{\delta }}\mathscr {L}(f_\mathscr {T}(\boldsymbol{x}+\boldsymbol{\delta }), y)), \end{aligned}$$

where $\nabla$ and sign are the vector differential and element-wise sign operators, respectively. To further degrade the model performance, Madry et al.^25^ proposed PGD that improved^24^ with multi-step optimization as follows:

5 $$\begin{aligned} \boldsymbol{\delta }^{s} = \Pi _{\varepsilon } [\boldsymbol{\delta }^{s-1} + \alpha \cdot \texttt {sign}(\nabla _{\boldsymbol{\delta }}\mathscr {L}(f_\mathscr {T}(\boldsymbol{x}+\boldsymbol{\delta }^{s-1}), y))], \end{aligned}$$

where $\Pi _\varepsilon$ is a projection function onto the $\varepsilon$-ball, and $\alpha$ is the step size. The initial perturbation $\boldsymbol{\delta }^0$ is a random vector and the resulting perturbation $\boldsymbol{\delta }$ corresponds to $\boldsymbol{\delta }^S$ for the predefined number of steps, *S*. Several studies have demonstrated that these simple attacks can effectively hinder deep learning models in various domains^47,48^.

However, these attacks, FGSM and PGD, require the model information to minimize Eqs. (4) or (5) because they assumed the *white-box setting* that an attacker has complete knowledge of the target system, including the model information. This assumption is unrealistic because deep learning models used for real-world systems, including BMSs, are generally utilized in private servers. That is, the target model $f_\mathscr {T}$ is kept securely inaccessible, preventing attackers from obtaining its gradients. Thus, the *black-box setting*, where the target models are inaccessible, is more practical for assessing the actual risk of adversarial examples, as it mirrors real-world deployment conditions^17^.

Under the black-box setting, the transfer attack, which generates adversarial examples with a source model that can be easily accessible by attackers, is regarded as the most efficient approach that leads to the misprediction of deep learning models. Formally, the adversarial example $\tilde{\boldsymbol{x}}$ is learned from the source model $f_{\mathscr {S}}$ as follows:

6 $$\begin{aligned} \tilde{\boldsymbol{x}} = \boldsymbol{x}+\max _{\Vert \boldsymbol{\delta }\Vert \le \varepsilon }\mathscr {L}(f_\mathscr {S}(\boldsymbol{x}+\boldsymbol{\delta }), y). \end{aligned}$$

Then, the generated example is used to deceive the target model into predicting an incorrect class ($f_\mathscr {T}(\tilde{\boldsymbol{x}}) \ne y$). Through this approach, the attacker can induce a meaningful performance drop in the unknown target model $f_{\mathscr {T}}$ by applying the standard gradient-based attacks on the source model $f_\mathscr {S}$^47,49^.

Nevertheless, under the black-box setting, the standard gradient-based attacks, such as FGSM and PGD, tend to overestimate the actual robustness of models because they do not utilize domain-specific knowledge that can capture unique model-independent data characteristics. Thus, several attacks with domain-specific knowledge have emerged to obtain transferable adversarial examples^22,23,26,50^. For example, Xie^26^ proposed the diverse input method (DIM) that generates adversarial examples with visual transformations during optimization by demonstrating that the transformations can significantly degrade the model performance in computer vision. Given a visual transformation $\phi$, DIM learns the perturbation $\boldsymbol{\delta }$ as follows:

7 $$\begin{aligned} \boldsymbol{\delta }^{s} = \Pi _\varepsilon [\boldsymbol{\delta }^{s-1} + \alpha \cdot \texttt {sign}(\nabla _{\boldsymbol{\delta }}\mathscr {L}(f_\mathscr {S}(\phi (\boldsymbol{x}+ \boldsymbol{\delta }^{s-1})), y))]. \end{aligned}$$

In addition, Dong et al.^50^ introduced the translation-invariant method (TIM) and showed that incorporating domain-specific knowledge can further improve the evaluation of the target models’ robustness in black-box settings. However, they were developed in the computer vision domain and were not inherently designed to handle time-series data, such as voltage signals of LIBs. While Kim et al.^23^ and Lee et al.^22^ developed adversarial attack methods that leverage frequency information for time-series data, their effectiveness in BMSs remains limited because both methods are designed for stationary vibration signals from rotating machinery, making them less effective when applied to the non-stationary nature of voltage signals in LIBs.

In contrast, our method significantly degrades SOH diagnosis performance in the black-box setting by explicitly accounting for the non-stationarity of voltage signals and selectively leveraging frequency information of voltage signals, which is the domain-specific knowledge beneficial for identifying each life stage of LIBs.

## Proposed method

**Fig. 2:**
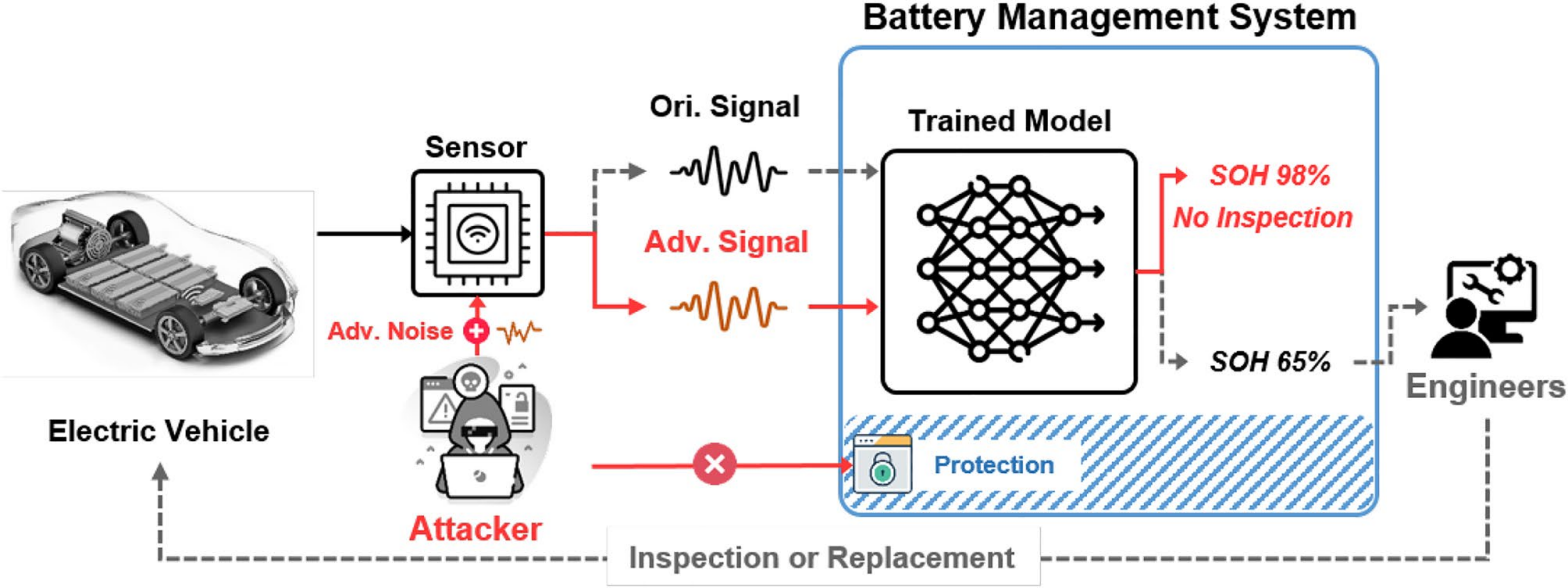
Illustration of the simulated practical BMS environment for SOH diagnosis. Since real-world BMSs are protected and obstruct attackers’ direct access to the trained model, white-box attacks are generally impossible, so attackers deceive the target model in the black-box setting.

### Problem statement

Figure 2 illustrates the main problem addressed in this study. The voltage signals obtained from LIBs are transmitted to a BMS, which is designed to ensure the safety and reliability of LIBs. Within the BMS, a predictive model analyzes the received signals to determine the current life stage of LIBs, enabling engineers to make timely inspections or replacements. However, attackers aim to compromise this model, referred to as a target model, within the BMS. Due to stringent security measures, it is difficult to access the model directly, making white-box attacks infeasible. Thus, they should consider black-box attacks in which no prior knowledge of the target model is available. In this scenario, the attackers create perturbations using an accessible source model and inject them into the original signals. As a result, the target model mispredicts the current SOH, potentially leading to incorrect maintenance decisions, safety risks, or significant financial losses.

To assess the practical risks of adversarial attacks on BMSs, we propose a novel transfer attack leveraging domain-specific knowledge, which can be effective in the black-box setting, as it exploits model-independent data characteristics. Let $\boldsymbol{x}\in \mathbb {R}^t$ be a voltage signal with the length *t*, and *y* be the label indicating the life stage of $\boldsymbol{x}$. We aim to learn a malicious but subtle perturbation $\boldsymbol{\delta }$ using a source model $f_\mathscr {S}$ to generate transferable adversarial example $\tilde{\boldsymbol{x}}=\boldsymbol{x}+\boldsymbol{\delta }$, inducing a target model $f_\mathscr {T}$ to deceive *y* as the other life stage $\tilde{y}$ ($\tilde{y} \ne y$).

### Importance of low-frequency information

To leverage the frequency information, previous studies have employed various frequency transformations for the waveforms of signals^51,52^. Since the voltage signals of LIBs are strongly non-stationary^53^, the discrete wavelet transform (DWT), one of the waveform analysis techniques, is effective for analyzing them as providing superior time-frequency localization with multiple resolutions^54^. Specifically, the DWT with *L* can decompose a signal $\boldsymbol{x}$ into a linear combination of wavelets with a series of low-pass and high-pass filters as follows:

8 $$\begin{aligned} \texttt {DWT}(\boldsymbol{x}) = \sum _{k=-\infty }^{\infty } a_k\varphi _{Lk}(x) + \sum _{l=1}^L \sum _{k=-\infty }^{\infty } b_{lk}\psi _{lk}(x), \end{aligned}$$

where $\varphi _{Lk}$ is a scaled and translated version of a wavelet $\varphi$ related to the low-pass filters ($\varphi _{Lk}=\varphi (2^L \boldsymbol{x}-k)$), and $\psi _{lk}$ is generated by dilations and shifts of a wavelet $\psi$ related to the high-pass filters ($\psi _{lk}=\psi (2^l \boldsymbol{x}-k)$). Here, $a_k$ is an *approximation* coefficient vector, and $b_{lk}$ is a *detail* coefficient vector when the decomposition level is *l*. The wavelets corresponding to the low-pass and high-pass filters are convolved with the signal $\boldsymbol{x}$; hence, the approximation coefficient vector typically captures low-frequency information of the signal, whereas the detail ones identify its high-frequency information^55^.

**Fig. 3:**
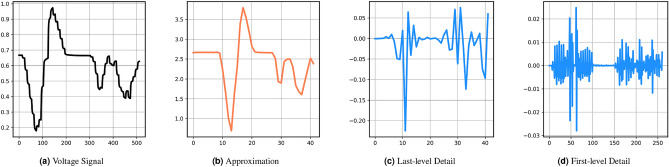
Examples of a voltage signal and its coefficients obtained by DWT with $L=4$. The approximation coefficient vector represents the overall temporal variations of the signal.

As shown in Fig. 3, low-frequency components, corresponding to the approximation coefficient vector, capture the overall temporal pattern of the signal, such as trend and periodicity, whereas high-frequency ones correspond to rapid changes or noises. Therefore, low-frequency information can provide the gradual degradation of LIBs over time, allowing for accurate and reliable assessments of their SOHs^30,31^.

Motivated by the impact of low-frequency information, we introduced a novel transfer attack that explicitly distorts the low-frequency components of the signals as well as their waveforms. The proposed method creates adversarial examples that pose risks to BMSs, assessing the practical robustness of SOH diagnosis models in BMSs. In the subsequent section, we describe its detailed procedure.

### Low-frequency aware transfer attack

**Fig. 4:**
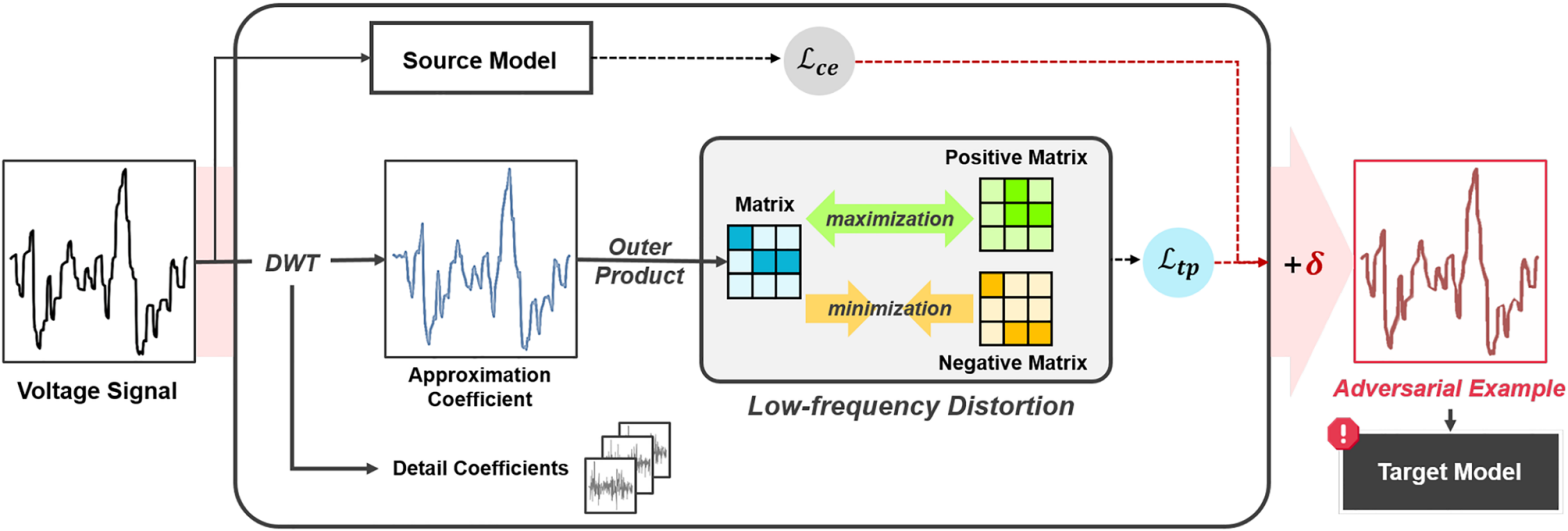
Overview of the proposed method.

We suggest a novel adversarial attack, *Low-frequency Aware Transfer Attack* (LATA), that manipulates the low-frequency information extracted from voltage signals along with their waveforms. LATA generates transferable adversarial examples that can severely threaten BMSs by capturing the gradual degradation patterns of LIBs under the black-box setting. Figure 4 shows an overview of our method.

Let $\boldsymbol{x}\in \mathbb {R}^t$ be a voltage signal with *t* observations, and *y* be a label corresponding to the life stage of $\boldsymbol{x}$. We learn a perturbation $\boldsymbol{\delta }$ to generate an adversarial example for $\boldsymbol{x}$ by distorting its *waveform* and *low-frequency*.

#### Waveform distortion

The waveform of $\boldsymbol{x}$ is corrupted with the cross-entropy loss function as the standard gradient-based methods, including FGSM^24^ and PGD^25^. Given a source model $f_\mathscr {S}$, the cross-entropy loss $\mathscr {L}_{ce}$ between a true class *y* of $\boldsymbol{x}$ and the predicted class $f_\mathscr {S}(x+\boldsymbol{\delta })$ is calculated as follows:

9 $$\begin{aligned} \mathscr {L}_{ce} = -y \log (f_\mathscr {S}(\boldsymbol{x}+\boldsymbol{\delta })). \end{aligned}$$

Maximizing this loss makes the waveform of $\boldsymbol{x}$ differ from its temporal pattern corresponding to *y*^26^.

#### Low-frequency distortion

If we only distort the waveforms, the resulting adversarial examples may overfit the source model $f_\mathscr {S}$ because the unique characteristics corresponding to each life stage of LIBs, which are independent of the model, cannot be reflected; thereby, the actual robustness of a target model $f_\mathscr {T}$ can be overestimated. That is, attacking voltage signals solely based on the waveform distortion may not sufficiently disrupt BMSs. Therefore, we also distort the low-frequency information of the signals, which involves their inherent characteristics that are useful for the SOH diagnosis, by leveraging the *negative triplet loss*.

Specifically, we first define the benign $\boldsymbol{x}$, which is not perturbed, as a positive sample $\boldsymbol{x}^+$ and a sample with the largest distance from $\boldsymbol{x}$ while belonging to a different class as a negative sample $\boldsymbol{x}^-$. Note that one can deliberately select the class which we desire to be misdiagnosed. Then, to utilize their frequency information, we decompose $\boldsymbol{x}$, $\boldsymbol{x}^+$, and $\boldsymbol{x}^-$ to their approximation and detail coefficients using the DWT.

As shown in Fig.  1b, when we directly distort the approximation coefficient values containing low-frequency information, the generated adversarial example exhibits an awkward pattern different from the original signal because the interactions between the coefficients can be overlooked. To be precise, since the approximation coefficient vector exhibits the overall temporal variations of the signals, directly manipulating them can cause unexpected changes in the waveforms of the resulting adversarial examples, leading to easy detection of whether it is attacked. Thus, to generate malicious but *imperceptible* examples that do not have awkward temporal variations, we consider relationships between the coefficients in the approximation coefficient vector as follows.

Let $\boldsymbol{a}$ be an approximation coefficient vector of $\boldsymbol{x}+\boldsymbol{\delta }$, and $\boldsymbol{a}^+$ and $\boldsymbol{a}^-$ be those of $\boldsymbol{x}^+$ and $\boldsymbol{x}^-$, respectively. We define their *interaction maps*, denoted as $\mathscr {A}$, $\mathscr {A}^+$, and $\mathscr {A}^-$, using the outer products of the corresponding approximation coefficient vectors with themselves as follows:

10 $$\begin{aligned} \mathscr {A} = \boldsymbol{a}\boldsymbol{a}^{\top }, \mathscr {A}^+ = \boldsymbol{a}^+ \boldsymbol{a}^{+\top }, \mathscr {A}^- = \boldsymbol{a}^- \boldsymbol{a}^{-\top }, \end{aligned}$$

where $\mathscr {A}$, $\mathscr {A}^+$, and $\mathscr {A}^-$ are matrices with the dimensions $|\boldsymbol{a}| \times |\boldsymbol{a}|$ such that $|\boldsymbol{a}|=|\boldsymbol{a}^+|=|\boldsymbol{a}^-|$. Following the properties of the outer product^56,57^, these interaction maps capture the structural information of each approximation coefficient vector ($\boldsymbol{a}$, $\boldsymbol{a}^+$, and $\boldsymbol{a}^-$) by encoding coefficient-wise energy through diagonal entries and pairwise interactions among low-frequency coefficients through off-diagonal entries^58–60^. Such interactions reflect how low-frequency components jointly determine the global trend and overall waveform shape of the corresponding signals ($\boldsymbol{x}+\boldsymbol{\delta }$, $\boldsymbol{x}^+$, and $\boldsymbol{x}^-$). By operating on the interaction maps rather than manipulating individual coefficients independently, the proposed method preserves the intrinsic correlation structure among low-frequency components, thereby maintaining the structural information of the signal and avoiding perceptually implausible distortions.

Subsequently, we use the negative triplet loss, $\mathscr {L}_{tp}$, to ensure that $\mathscr {A}$ becomes similar to $\mathscr {A}^-$ while simultaneously distancing from $\mathscr {A}^+$. Then, it can be formulated as follows:

11 $$\begin{aligned} \mathscr {L}_{tp} = - \max (\Vert \mathscr {A}-\mathscr {A}^-\Vert _2 - \Vert \mathscr {A}-\mathscr {A}^+\Vert _2 + \xi , 0), \end{aligned}$$

where $\xi$ is a non-negative margin, indicating the minimum acceptable distance between $\Vert \mathscr {A}$-$\mathscr {A}^+\Vert$ and $\Vert \mathscr {A}$-$\mathscr {A}^-\Vert$ pairs. By maximizing this loss, the low-frequency information of $\boldsymbol{x}$ becomes distant from that of the positive sample and similar to that of the negative one, taking into account the relations between the approximation coefficients. In other words, the overall temporal shape of $\boldsymbol{x}$ becomes similar to that of another life stage, not its original one, without awkward variations. Note that this formulation is compatible with realistic black-box settings, as the construction of the triplets relies solely on label information available in a surrogate (source) dataset accessible to the attacker.

**Algorithm 1:**
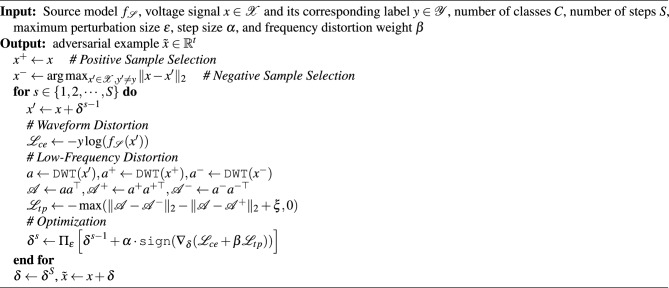
Low-frequency aware transfer attack

#### Multi-step optimization

Using these loss functions related to waveform and low-frequency distortions, we learn a malicious but subtle perturbation $\boldsymbol{\delta }$ within the maximum perturbation size $\varepsilon$. In particular, the perturbation $\boldsymbol{\delta }$ is learned by multi-step optimization^25^ with the number of steps *S* and step size $\alpha$. For each step $s \in \{1, 2, \cdots , S\}$, the perturbation $\boldsymbol{\delta }^{s}$ is calculated as follows:

12 $$\begin{aligned} \boldsymbol{\delta }^{s} = \Pi _\varepsilon \left[ \boldsymbol{\delta }^{s-1} + \alpha \cdot \texttt {sign}(\nabla _{\boldsymbol{\delta }} (\mathscr {L}_{ce} + \beta \mathscr {L}_{tp})) \right] , \end{aligned}$$

where $\Pi _\varepsilon$ is a projection function onto the $\varepsilon$-ball and $\beta$ is a weight for the low-frequency distortion. Note that the initial perturbation $\boldsymbol{\delta }^0$ is a random vector, and the perturbation $\boldsymbol{\delta }^S$ in the last step *S* is regarded as the final perturbation $\boldsymbol{\delta }$.

By adding the learned perturbation $\boldsymbol{\delta }$ to the original voltage signal $\boldsymbol{x}$, we can obtain an adversarial example $\tilde{\boldsymbol{x}}$ ($\tilde{\boldsymbol{x}}=\boldsymbol{x}+ \boldsymbol{\delta }$). The overall procedure for generating adversarial examples is summarized in algorithm 1.

## Experiments

In the experiments, we considered two practical scenarios for the adversarial attacks against BMSs: *random* and *targeted*. On the one hand, in the *random attack* scenario, adversarial examples are generated to make the life stage assigned to each signal perceived as another arbitrary stage. This scenario is similar to the practical situation in which an attacker corrupts a signal with the normal stage to create a signal with an arbitrary degradation stage, resulting in opportunity costs due to unnecessary maintenance. On the other hand, for the *targeted attack* scenario, adversarial examples are generated so that the original life stage of each signal is deliberately misperceived to a specific stage. This can be regarded as a real-world situation in which a signal in a degraded stage is attacked to be diagnosed as the normal one, causing severe accidents that harm the safety of users or employees. Thus, we performed various experiments to demonstrate that the proposed method generates malicious but imperceptible signals by learning effective perturbations to hinder SOH diagnosis models in BMSs in both attack scenarios.

### Experimental settings

*Dataset* To validate the effectiveness of our method in evaluating adversarial robustness, we employed the LG18650HG2 LIB dataset^32^. This dataset collected the voltage, current, temperature, and capacity of LIBs in electric vehicles over time in various driving environments at different temperatures. In particular, the dataset consists of driving data for 12 electric vehicles under six external temperatures, − 20, − 10, 0, 10, 25, and 40 °C, and four driving environments, namely UDDS, HWFET, US06, and LA92. Here, we regarded the dataset that randomly mixed four driving environments at the external temperature of 0 °C as $\mathscr {X}$.

**Fig. 5:**
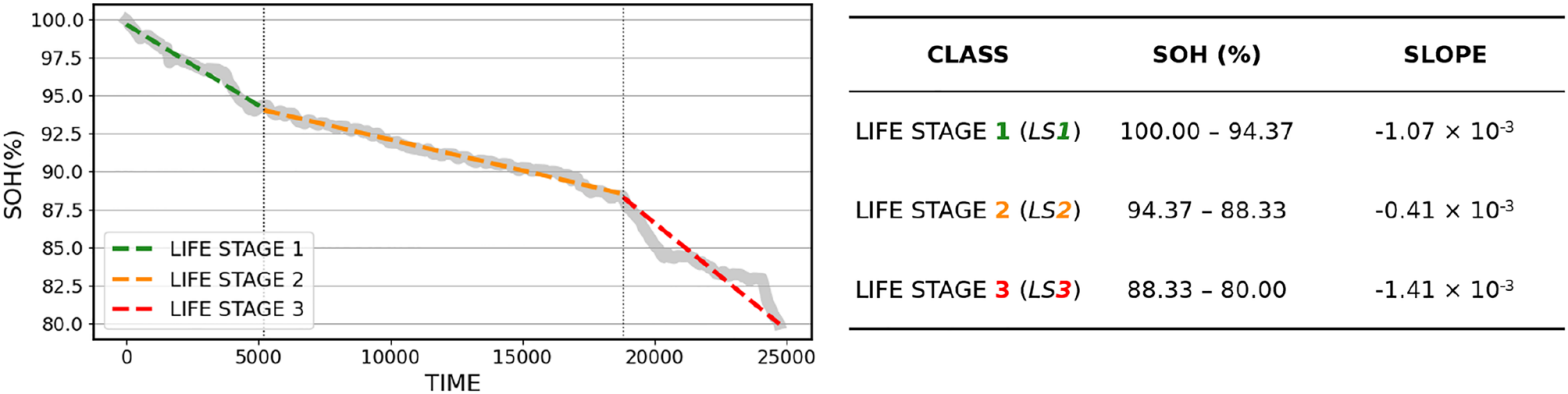
Life stages depending on the SOH values of the dataset.

*Data preprocessing* We first computed the SOH corresponding to each voltage signal $\boldsymbol{x}\in \mathscr {X}$ with the length $t=512$ using Eq.  (1). The SOH values were subsequently categorized into three distinct life stages, as illustrated in Fig.  5. Following Dubarry et al.^45^ and Fan et al.^46^, we demarcated the life stages based on two specific timestamps that occur salient shifts in the degradation trends of SOHs. The first life stage (*LS1*) was assigned to the signals with SOH values ranging from 100.00 to 94.37%, characterized by a degradation trend line slope of $-1.07\times 10^{-3}$. The second and third life stages (*LS2* and *LS3*) were allocated to the signals with SOH values ranging from 94.37 to 88.33% and from 88.33 to 80.00%, respectively, each associated with degradation trend line slopes of $-0.41\times 10^{-3}$ and $-1.41\times 10^{-3}$, respectively. Next, we normalized all signals $\boldsymbol{x}\in \mathscr {X}$ to the range $[-1, 1]$ using

13 $$\begin{aligned} \bar{\boldsymbol{x}} = 2 \times (\boldsymbol{x}- \boldsymbol{x}_{min})/(\boldsymbol{x}_{max}-\boldsymbol{x}_{min}) - 1, \end{aligned}$$

where $\bar{\boldsymbol{x}}$ denotes the normalized signal, and $\boldsymbol{x}_{max}$ and $\boldsymbol{x}_{min}$ represent the maximum and minimum values within $\boldsymbol{x}$, respectively. We constructed a dataset for the SOH diagnosis task for LIBs. Among 24,210 sequences in the dataset, we used 80% of them for training SOH diagnosis models, 10% for validation, and the remaining 10% for evaluating the model performance.

*Model architectures* We evaluated a diverse set of SOH diagnosis models spanning structurally distinct architectures, covering feed-forward, recurrent, convolutional, and Transformer-based designs. Specifically, we selected the multilayer perceptron (MLP)^61^ as a feed-forward baseline; LSTM^62^ and the gated recurrent units (GRU)^63^ to capture recurrent temporal dependencies; the temporal convolutional network (TCN)^64^, the deep convolutional neural network with wide first-layer kernels (WDCNN)^65^, and the one-dimensional CNN (CNN1D)^66^ to extract local and hierarchical patterns; and Informer^67^ as a representative Transformer-based architecture for long-sequence modeling. These models have been widely adopted in the literature for battery SOH estimation and time-series modeling^68–74^. Evaluating the proposed method across this diverse set of architectures enables a comprehensive assessment of its generality and transferability.

*Baseline methods* Since transfer attacks specifically designed for SOH diagnosis models in BMSs have been largely unexplored, we adopted DIM^26^ and TIM^50^, which have demonstrated their transferability in various domains, as well as the popular adversarial attacks, FGSM^24^ and PGD^25^, as baselines to evaluate the attack performance of the proposed method.

*Hyperparameters* The default settings were used for the architectures of the baselines^61–67^. For all baselines and our method, the number of steps *S* was set to $10^1$, and the maximum perturbation size $\varepsilon$ was fixed at $10^{-1}$, with the step size $\alpha$ defined as $2\varepsilon /S$. For our approach, LATA, we employed the Daubechies 6 (db6) wavelet for the DWT. This wavelet was chosen for its practical balance between time-frequency localization and smoothness, which is well suited for decomposing LIB voltage signals^29,30,53,55,75^. Given that LIB voltage trajectories are typically non-stationary yet smoothly varying, db6 can effectively capture low-frequency degradation trends while suppressing high-frequency noise. The decomposition level was set to $L=3$. In addition, we set the frequency distortion weight $\beta$ to $10^1$ and the margin $\xi$ for the negative triplet loss function to 1. A sensitivity analysis for each hyperparameter is provided in Sensitivity Analysis.

*Evaluation metric* To measure the SOH diagnosis performance, we used the macro-average F1 score, which can consider the imbalance between the number of samples belonging to each life stage.

*Computational resources* All experiments were executed using the deep learning framework PyTorch (version 1.13.0, available at https://pytorch.org/) on a system with an Intel Core i9-10900X CPU clocked at 3.70 GHz, 256 GB RAM, and NVIDIA GeForce RTX 3090 24GB GPU. Data processing and analysis were performed using NumPy (version 1.26.0, available at https://numpy.org/) and Pandas (version 2.1.1, available at https://pandas.pydata.org/), and all figures were generated using Matplotlib (version 3.8.0, available at https://matplotlib.org/).

### Experimental results

We first examined the efficacy of the low-frequency distortion that leverages the interaction maps, which is the main contribution of our work. Then, we assessed the transfer attack performance of LATA against SOH diagnosis models compared to those of the baselines. Finally, a series of sensitivity analyses were conducted to evaluate the impact of the hyperparameters used in the proposed method.

Table 1 presents the undisturbed macro-averaged F1 scores of SOH diagnosis models designed with seven different architectures. They performed extremely well in correctly diagnosing the life stages of almost all signals.

**Table 1:** Undisturbed macro-averaged F1 scores for seven SOH diagnosis models.

MLP	TCN	LSTM	GRU	WDCNN	CNN1D	Informer
1.0000	1.0000	0.9995	0.9998	1.0000	1.0000	1.0000

#### Effect of low-frequency distortion

**Fig. 6:**
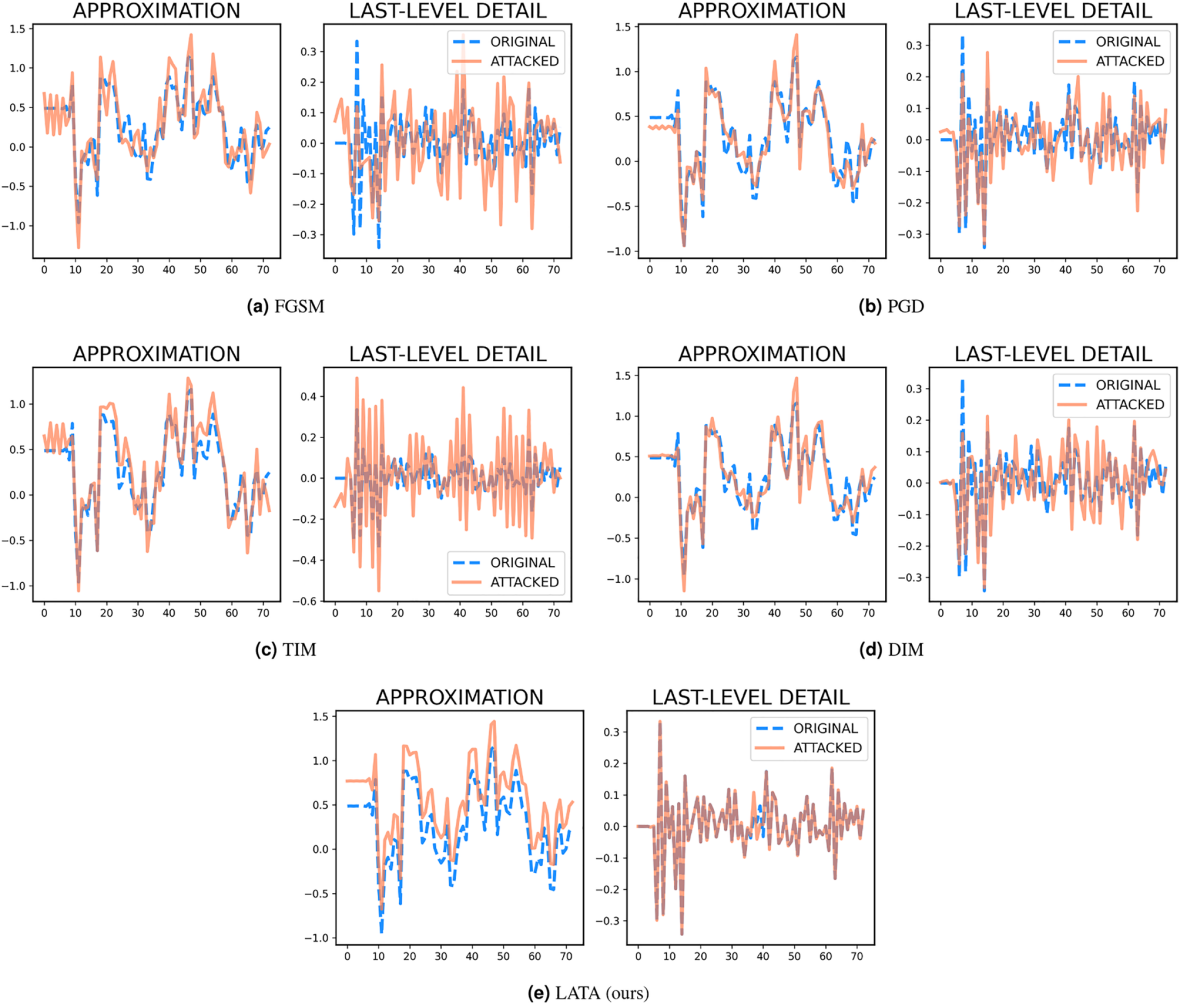
Comparison of approximation and last-level detail coefficients derived by DWT with $L=3$ for the original signal with those for adversarial examples from (**a**) FGSM, (**b**) PGD, (**c**) TIM, (**d**) DIM, and (**e**) LATA.

To investigate the effectiveness of low-frequency distortion in our approach, we first compared the approximation and detail coefficients of adversarial examples obtained from LATA with those from the baselines in the random attack scenario. Here, we used CNN1D as the source model for generating adversarial examples. As shown in Fig. 6a–d, the baselines predominantly corrupted the detail coefficients, which involve noises from various sources with no significant impact on SOH, rather than the approximation ones. In other words, the existing methods learned the perturbation without consideration of the domain knowledge, so they are inappropriate to hinder SOH diagnosis. By contrast, as in Fig.  6e, LATA thoroughly focused on distorting the approximation coefficients, which capture the overall signal shape, being important to estimate SOH.

Then, we verified the impact of *interaction map* in the low-frequency distortion, which helps generate imperceptible adversarial examples. Figure 1 illustrates approximation coefficient vectors and voltage signals obtained from LATA and its ablation method, *LATA w/o IM*, which directly distorts the approximation coefficient values instead of utilizing the interaction map. We observed that the interaction map enabled us to obtain the approximation coefficient vector with temporal variation patterns similar to the original one by preserving the relations between each coefficient. Thus, it is hard to detect whether the adversarial example in Fig.  1a has been attacked because it does not have awkward temporal variations caused by meaningless noises that occurred in Fig.  1b.

**Fig. 7:**
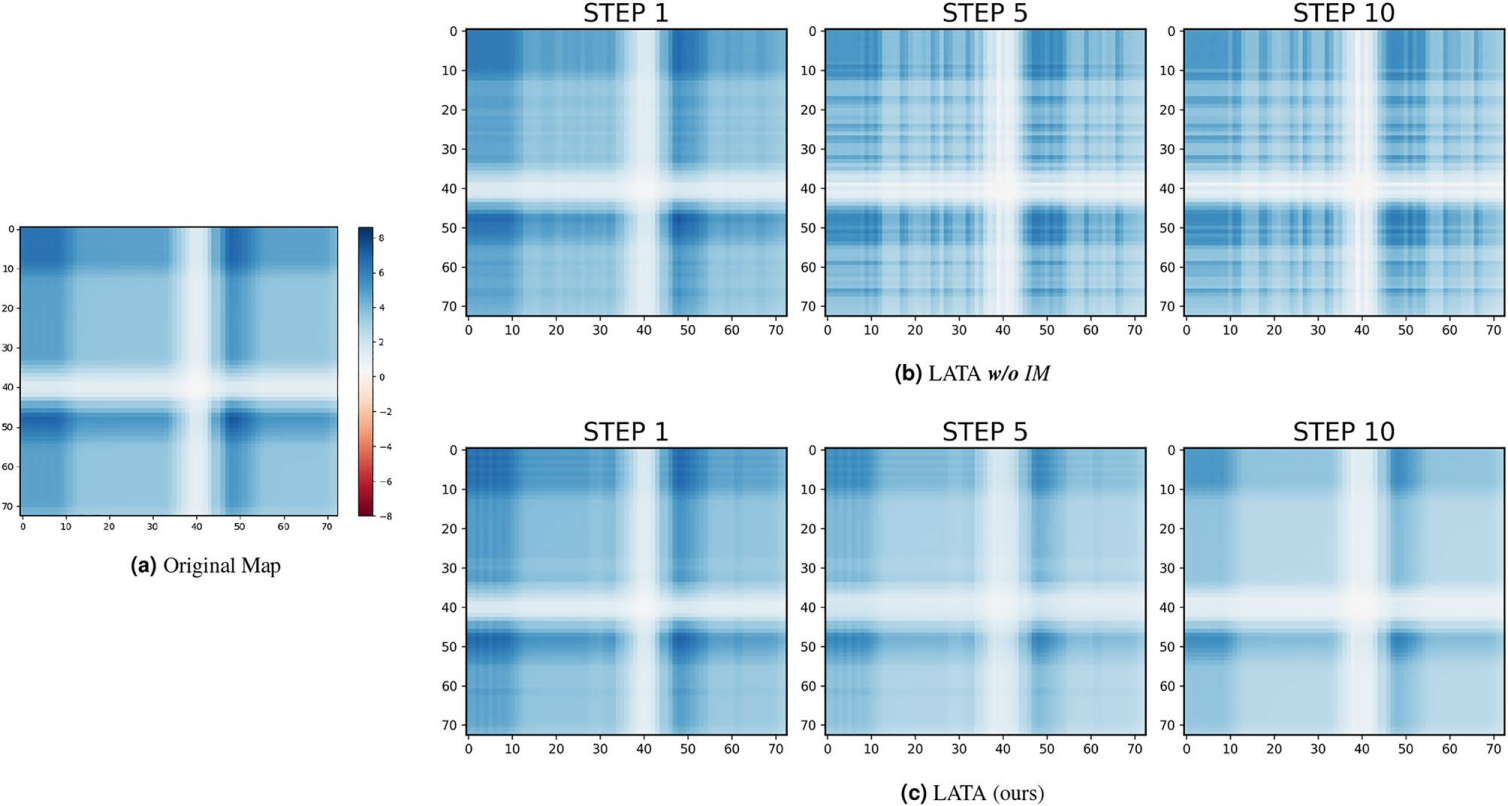
Comparison of the interaction maps of (**a**) original approximation coefficients with those obtained from adversarial examples generated by (**b**) LATA *w/o IM* and (**c**) LATA across steps ($S=10$) under the random attack scenario.

Moreover, as in Fig.  7, we visualized the interaction maps, which indicate the structural information of approximation coefficient vectors derived from LATA and *LATA w/o IM* during the multi-step optimization ($S=10$). While the structural information from *LATA w/o IM* became notably distant from the original one during optimization, the proposed method thoroughly maintained the relations of the original approximation coefficients at every step. Thus, we reaffirmed that the interaction map successfully preserves the structural relations in the original approximation coefficient vector.

**Fig. 8:**
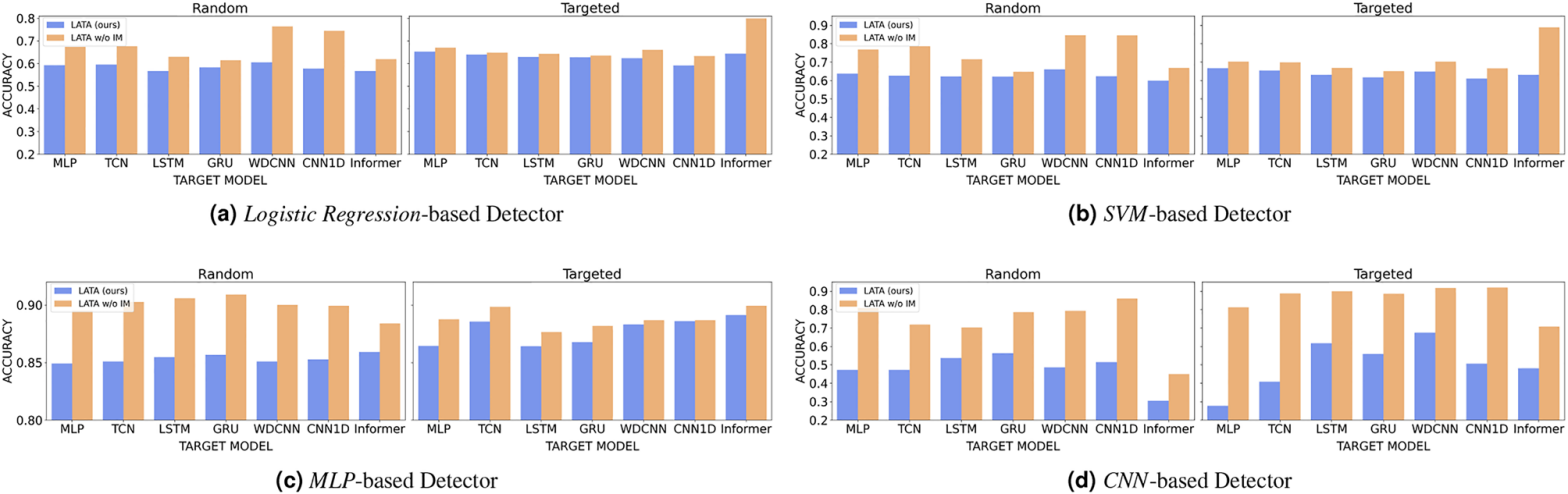
Detection performance of adversarial examples generated by LATA and *LATA w/o IM* across seven source models using (**a**) *Logistic Regression*- (**b**) *SVM*- (**c**) *MLP*-, (**d**) *CNN*-based detectors.

Subsequently, we evaluated the *imperceptibility* of adversarial examples obtained by LATA compared to *LATA w/o IM*. Following prior work, we employed four binary classifiers, logistic regression^48^, support vector machine (SVM)^76^, MLP^77,78^, and CNN^79^, as detectors to distinguish benign signals from adversarial ones. Here, we constructed a balanced dataset by combining original test samples with their corresponding adversarial counterparts. Each detector was trained on 90% of this dataset, while the remaining 10% was reserved for evaluation. Since the dataset was balanced, detection accuracy was used as the performance metric. As shown in Fig.  8, LATA consistently yielded lower detection performance than *LATA w/o IM* across all detectors under both random and targeted scenarios, indicating improved practical stealthiness. Notably, excluding the MLP-based detector, detection performance generally remained around or below 0.6, which is close to random guessing. Even with the MLP-based detector, LATA reduced detectability compared to *LATA w/o IM*, demonstrating the effectiveness of the interaction map.

**Table 2:** Comparison of SOH diagnosis performance for each model depending on the utilization of the interaction map.

Scenario	Interaction map	MLP	TCN	LSTM	GRU	WDCNN	CNN1D	Informer
Random	$\checkmark$	**0.5172**	0.5190	**0.5565**	**0.5643**	**0.5108**	**0.5720**	**0.5353**
	$\times$	0.5431	**0.5085**	0.6550	0.5940	0.5699	0.6625	0.6514
Targeted	$\checkmark$	**0.5162**	**0.5372**	**0.5551**	**0.5571**	**0.5177**	**0.5790**	**0.5298**
	$\times$	0.5527	0.5800	0.6034	0.6212	0.5713	0.6468	0.5911

Better attack performance (lower SOH diagnosis performance) is highlighted in boldface.

Furthermore, Table 2 exhibits the averaged SOH diagnosis performance across the target models on adversarial examples generated by LATA and *LATA w/o IM* based on each source model. LATA showed a relatively large performance drop compared to *LATA w/o IM* in most cases; thereby, the interaction map used in the low-frequency distortion successfully learns subtle perturbation that generates *malicious* but *imperceptible* examples, effectively hindering SOH diagnosis in BMSs.

#### Comparison with baselines

To validate the superiority of our method in evaluating the practical robustness of SOH diagnosis models in BMSs, we compared the attack performance of LATA with those of the baselines under the black-box setting. Table 3 shows the SOH diagnosis performance for each source-target model pair, reported as the macro-averaged F1 scores under both *random* and *targeted* attack scenarios.

**Table 3:** Macro-averaged F1 scores of target models evaluated on adversarial examples generated from source models by different attack methods.

Model		*Random* attack scenario					*Targeted* attack scenario				
Source	Target	FGSM	PGD	TIM	DIM	**LATA**	FGSM	PGD	TIM	DIM	**LATA**
MLP	TCN	0.5849	0.5320	**0.4816**	0.5370	0.5719	0.5915	0.6276	0.5751	0.6673	**0.5543**
	LSTM	0.5129	0.4930	0.6057	0.5885	**0.4351**	0.4769	0.5154	0.5161	0.5819	**0.4503**
	GRU	0.5253	0.5061	0.5055	0.5304	**0.4501**	0.4624	0.5098	0.5174	0.5785	**0.4319**
	WDCNN	0.6252	0.6032	**0.5897**	0.6048	0.6886	**0.6326**	0.6520	0.6747	0.7374	0.6470
	CNN1D	0.5291	0.5455	0.6183	0.6288	**0.4030**	0.4979	0.5183	0.5303	0.5216	**0.4701**
	Informer	0.5773	0.5616	0.6162	0.7089	**0.5545**	0.5739	0.6062	0.5909	0.6687	**0.5434**
TCN	MLP	0.6398	0.6621	0.6883	0.6389	**0.6306**	0.7341	0.7546	0.7989	0.7632	**0.6959**
	LSTM	**0.4248**	0.4839	0.5445	0.5004	0.4322	0.4752	0.5450	0.6621	0.5912	**0.4377**
	GRU	**0.4309**	0.4814	0.5488	0.4876	0.4411	0.5033	0.5339	0.6738	0.5704	**0.4256**
	WDCNN	0.5221	0.5460	0.5877	**0.5011**	0.6551	**0.5732**	0.6051	0.7364	0.6125	0.6545
	CNN1D	0.5918	0.5351	0.6191	0.6298	**0.4062**	0.5479	0.5577	0.6322	0.5923	**0.4641**
	Informer	0.6447	**0.5225**	0.5700	0.6268	0.5486	0.5615	0.5755	0.5922	0.6096	**0.5453**
LSTM	MLP	0.9074	0.9194	0.8014	0.9192	**0.6785**	0.8844	0.9561	0.9078	0.9549	**0.7558**
	TCN	0.8796	0.8846	0.7026	0.8806	**0.6371**	0.8334	0.9504	0.8420	0.9484	**0.6575**
	GRU	0.4416	0.0498	0.1793	**0.0364**	0.3235	0.3912	**0.0337**	0.3054	0.0362	0.1663
	WDCNN	0.8740	0.9109	0.7894	0.9098	**0.6979**	0.8398	0.9767	0.9180	0.9686	**0.7209**
	CNN1D	0.5191	0.5552	0.5899	0.5565	**0.4078**	0.4842	0.5329	0.5116	0.5246	**0.4504**
	Informer	0.6820	0.7310	0.7865	0.7309	**0.5943**	0.6584	0.8146	0.7684	0.8218	**0.5796**
GRU	MLP	0.8703	0.9070	0.8657	0.9086	**0.6996**	0.8730	0.9747	0.9115	0.9699	**0.7259**
	TCN	0.7877	0.8272	0.7380	0.8111	**0.6294**	0.7689	0.9158	0.8163	0.9128	**0.6334**
	LSTM	0.3284	**0.0260**	0.1315	0.0284	0.3552	0.3366	0.1278	0.3602	**0.1049**	0.2654
	WDCNN	0.7835	0.8583	0.8297	0.8147	**0.7096**	0.7653	0.9151	0.8966	0.9167	**0.6999**
	CNN1D	0.4919	0.4853	0.4685	0.4851	**0.4007**	0.4723	0.5221	0.4945	0.5092	**0.4613**
	Informer	0.6001	0.6744	0.6246	0.6742	**0.5915**	0.5910	0.8207	0.7211	0.8130	**0.5569**
WDCNN	MLP	0.8876	0.8691	0.6633	0.8327	**0.6449**	0.9033	0.9146	0.7597	0.8831	**0.6914**
	TCN	0.7309	0.7478	**0.4496**	0.6871	0.5766	0.7766	0.8260	**0.5369**	0.7837	0.5725
	LSTM	0.5448	0.5955	0.4896	0.5700	**0.4336**	0.5770	0.6450	0.5596	0.6139	**0.4280**
	GRU	0.5007	0.5665	0.4684	0.5258	**0.4464**	0.5360	0.6221	0.5329	0.5847	**0.4272**
	CNN1D	0.4723	0.4653	0.5064	0.4806	**0.4026**	0.4787	0.4764	0.5485	0.4820	**0.4481**
	Informer	0.6466	0.6373	**0.4287**	0.6205	0.4812	0.6671	0.6754	0.5297	0.6817	**0.4706**
CNN1D	MLP	0.9534	0.9807	0.8236	0.9875	**0.6692**	0.9160	0.9869	0.8763	0.9911	**0.7481**
	TCN	0.9296	0.9629	0.6478	0.9544	**0.5953**	0.8770	0.9779	0.7314	0.9728	**0.6620**
	LSTM	**0.3814**	0.4773	0.4917	0.4692	0.4359	**0.3646**	0.5022	0.5782	0.4884	0.4190
	GRU	0.3749	0.3566	0.4826	**0.3391**	0.4439	**0.3590**	0.3945	0.5742	0.3844	0.3796
	WDCNN	0.8410	0.8960	0.7620	0.8755	**0.6938**	0.7975	0.9122	0.8413	0.8840	**0.6975**
	Informer	0.7231	0.8665	0.6430	0.8618	**0.5936**	0.6805	0.8609	0.7107	0.8470	**0.5676**
Informer	MLP	0.7458	0.7301	0.7085	0.7550	**0.6526**	0.7877	0.7936	0.8058	0.8088	**0.6576**
	TCN	0.5371	0.4958	**0.4125**	0.4887	0.5349	0.5678	0.5774	0.5207	0.5560	**0.5125**
	LSTM	**0.3574**	0.4183	0.4739	0.4324	0.4337	**0.3848**	0.4769	0.5543	0.5002	0.4407
	GRU	**0.3830**	0.3930	0.4761	0.4148	0.4464	**0.4045**	0.4419	0.5418	0.4857	0.4340
	WDCNN	0.6191	0.6274	0.6193	**0.6057**	0.6373	0.6372	0.6738	0.7179	0.6600	**0.6079**
	CNN1D	0.5534	0.5789	0.6060	0.6055	**0.5068**	0.5696	0.6015	0.6716	0.6317	**0.5261**
*Average score*		0.6206	0.6211	0.5880	0.6277	**0.5393**	0.6174	0.6670	0.6565	0.6739	**0.5417**
*# of largest drop*		5	2	5	4	**26**	6	1	1	1	**33**

The best attack performance (the lowest F1 score) across attack methods for each source-target pair is highlighted in boldface.

In the random attack scenario, LATA consistently outperformed all baseline methods regardless of the source models. Specifically, the proposed method achieved an average score of 0.5393, outperforming the second-best baseline, TIM, by about 0.05 and the worst baseline, FGSM, by about 0.08. This consistent performance drop across diverse source models indicates that LATA effectively uncovers the practical adversarial robustness of SOH diagnosis models in BMSs by exploiting frequency-domain information to learn malicious perturbations.

In the targeted attack scenario, the proposed method also outperformed the existing attack methods. In this setting, signals in the normal stage (*LS1*) were targeted toward degradation stages (*LS2* or *LS3*), while signals in degradation stages were reassigned to the normal stage. Notably, in 33 out of 42 cases, LATA achieved the largest performance drop, with an average score of 0.5417 for SOH diagnosis. The performance gap was particularly pronounced when the target model was MLP, where LATA induced substantial degradation regardless of the source model, whereas other attack methods led to only marginal drops.

The results in Table 3 further provide insights from three complementary perspectives. First, in terms of source–target transferability, LATA consistently yielded larger performance drops across diverse source–target pairs, suggesting that the proposed low-frequency manipulation captures model-independent characteristics rather than source-specific artifacts. Second, with respect to architecture-dependent vulnerability, MLP- and Transformer-based target models exhibited more severe degradation under LATA, indicating that models relying heavily on global temporal patterns are particularly sensitive to structured low-frequency distortions. Third, when comparing random and targeted attack scenarios, LATA maintained strong and consistent attack performance in both settings, whereas baseline methods often exhibit more variable behavior depending on the attack objective. These findings validate the effectiveness of LATA for evaluating the real-world robustness of BMSs.

**Fig. 9:**
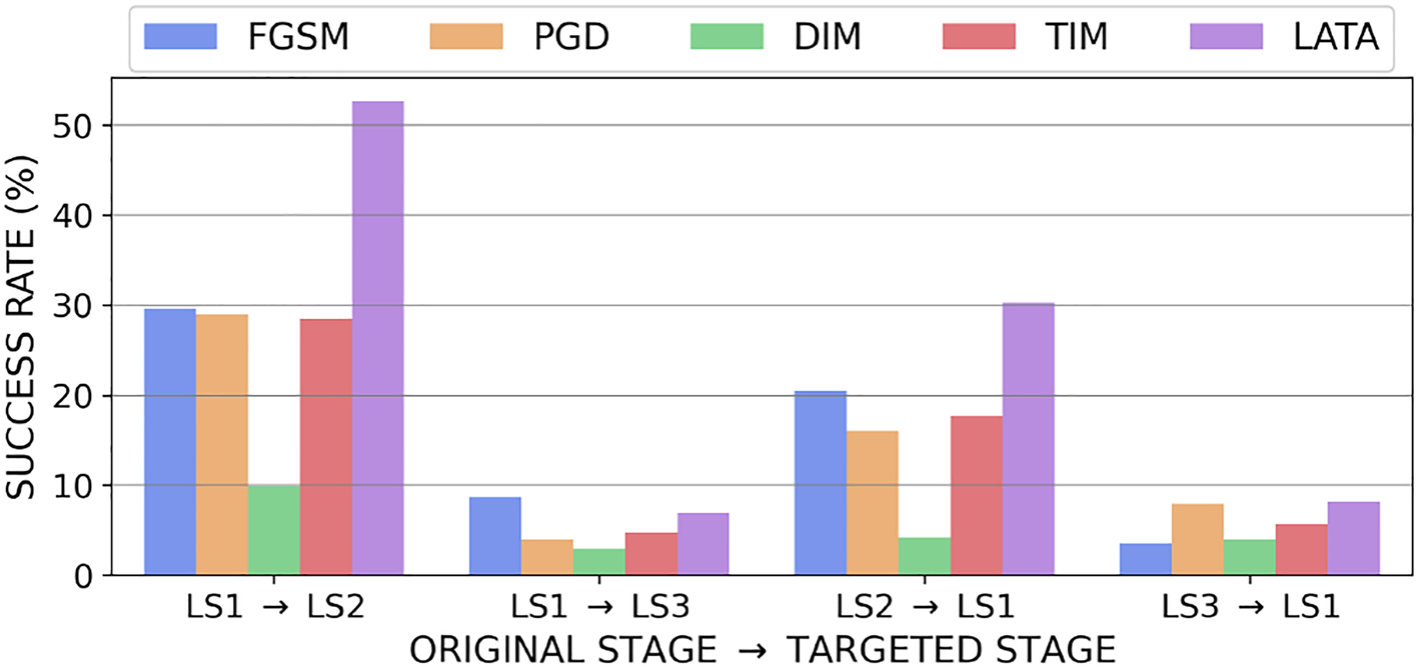
Transfer attack success rates of LATA and baselines.

We further investigated the practicability of LATA in the targeted attack scenario by measuring the transfer attack success rates and comparing them to those of the baselines. Here, we presented the averaged success rates across source models for each attack method. As summarized in Fig.  9, the proposed method generally exhibited superior transfer attack success rates both when perturbing *LS1* to *LS2* and the opposite cases, which are regarded as important diagnostic points for optimal LIB management. When inducing confusion from *LS1* to *LS3* and the opposite cases, all methods, including LATA, showed low attack success rates given a reasonable perturbation size. Such low performance occurs because *LS3* is more deteriorated than *LS1* and *LS2*, making limited perturbations alone insufficient to effectively mislead the BMS.

#### Sensitivity analysis

We conducted a comprehensive sensitivity analysis of the hyperparameters used in LATA to understand their impact on the robustness evaluation of SOH diagnosis models. We investigated four hyperparameters: the maximum perturbation size $\varepsilon$, the number of steps *S*, the low-frequency distortion weight $\beta$, and the decomposition level *L*. For this analysis, we leveraged CNN1D, which showed decent attack performance in both scenarios, as the source model for generating adversarial examples for all baselines.

**Fig. 10:**
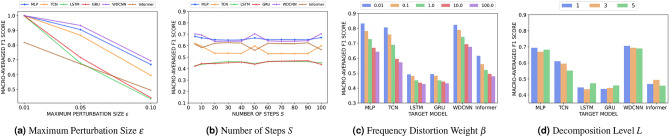
Macro-averaged F1 scores for six target models attacked by LATA with different values of (**a**) maximum perturbation size $\varepsilon$, (**b**) number of steps *S*, (**c**) frequency distortion weight $\beta$, and (**d**) decomposition level *L*.

Figure 10 aprovides the macro-averaged F1 scores of the six target models evaluated on adversarial examples derived by our method under different $\varepsilon$ values. As larger values of $\varepsilon$ allow stronger perturbations, they showed a higher performance drop in all cases. Based on this observation, we selected $\varepsilon =0.1$ as it yielded a clear and consistent performance degradation while remaining within a reasonable perturbation range.

In Fig. 10b, we analyzed the impact of *S* on the performance degradation by varying *S* from 5 to 100 in increments of 10. The results showed that attack performance largely saturated after approximately 10 steps, with additional iterations yielding only marginal improvements. Accordingly, we set $S=10$ to ensure computational efficiency without sacrificing attack effectiveness.

We further examined the effect of the low-frequency distortion weight ($\beta$), which balances waveform distortion and low-frequency manipulation. As shown in Fig. 10c, the performance of all target models monotonically decreased as $\beta$ increased, indicating that stronger distortion of low-frequency components led to more severe degradation in SOH diagnosis performance. We therefore set $\beta =10$, as this value allowed effective low-frequency distortion while avoiding excessive changes to the original waveform, resulting in strong attack performance.

Finally, we conducted a comparative analysis by varying the decomposition level (*L*). Figure 10d shows that model performance varied only slightly across different values of *L*, with most differences falling within a narrow range of 0.01–0.05 in macro-averaged F1 scores. Based on this analysis, we set $L=3$, which consistently provided stable attack performance across different target models while maintaining imperceptible distortions.

## Conclusion

We proposed a novel transfer attack method, LATA, designed to accurately evaluate the practical adversarial robustness of deep learning-based SOH diagnosis in BMSs. The proposed method jointly manipulates the waveforms of voltage signals from LIBs and their low-frequency information, which affects the overall signal shape, to generate adversarial examples that are malicious to BMS. Moreover, by explicitly modeling the relationships among low-frequency components through interaction maps, LATA mitigates unnatural temporal distortions and produces imperceptible adversarial perturbations.

Our experiments showed that LATA consistently achieves strong attack effectiveness in realistic black-box setting that closely reflects industrial BMS operations. These findings reveal an underexplored security risk: SOH diagnosis pipelines can be disrupted without white-box access by exploiting low-frequency structure in voltage signals. Consequently, our study highlights the need for systematic robustness assessment and principled defense mechanisms in industrial BMS deployments.

As directions for future work, we plan to relax the attacker’s assumptions to better reflect real-world deployment variability by considering additional forms of distribution shift, such as different signal window lengths, alternative normalization schemes, and cross-dataset evaluations (see *Supplementary Material* for preliminary results). In addition, while the current triplet-loss formulation leverages label information available in an attacker-controlled surrogate dataset during offline attack construction, we aim to develop label-free or weakly supervised alternatives, such as cluster-aware or self-supervised triplet selection in the low-frequency feature space, to further reduce reliance on labeled data. In parallel, we will investigate defense strategies to harden BMSs against low-frequency adversarial manipulation, advancing toward more trustworthy and robust BMSs.

## Data Availability

The dataset used in this study is publicly available at https://data.mendeley.com/datasets/cp3473x7xv/3

## References

[1] Pradhan, S. K. & Chakraborty, B. Battery management strategies: An essential review for battery state of health monitoring techniques. *J. Energy Storage* **51**, 104427 (2022).

[2] Zhang, S., Liu, Z. & Su, H. Battery early prognostics based on pseudo meta-learning. *IEEE Trans. Ind. Inf.* **20**(10), 11655–11665 (2024).

[3] Guo, Y., Yang, D., Zhang, Y., Wang, L. & Wang, K. Online estimation of soh for lithium-ion battery based on SSA-Elman neural network. *Protect. Control Modern Power Syst.* **7**, 40 (2022).

[4] Gu, X. et al. Early warning of thermal runaway based on state of safety for lithium-ion batteries. *Commun. Eng.* **4**, 1–9 (2025). 40494989 10.1038/s44172-025-00442-1 PMC12152129

[5] Gupta, C. & Farahat, A. Deep learning for industrial AI: Challenges, new methods and best practices. In *Proceedings of the 26th ACM SIGKDD International Conference on Knowledge Discovery & Data Mining*, 3571–3572 (2020).

[6] Lee, S. et al. Multi-order graph attention network for water solubility prediction and interpretation. *Sci. Rep.* **13**, 957 (2023). 36864064 10.1038/s41598-022-25701-5 PMC9981901

[7] Lee, S., Choi, J. & Son, Y. Efficient visibility algorithm for high-frequency time-series: Application to fault diagnosis with graph convolutional network. *Ann. Oper. Res.* **339**, 813–833 (2024).

[8] Haim, G., Martina, S., Howell, J., Bar-Gill, N. & Caruso, F. Machine-learning based high-bandwidth magnetic sensing. *Mach. Learn. Sci. Technol.* **6**(2), 025074 (2025).

[9] Zhang, Y. & Li, Y.-F. Prognostics and health management of lithium-ion battery using deep learning methods: A review. *Renew. Sustain. Energy Rev.* **161**, 112282 (2022).

[10] Weinreich, J., Karandashev, K., Arrieta, D. J. A., Hermansson, K. & von Lilienfeld, O. A. Calculated solvation and ionization energies for thousands of organic molecules relevant to battery design. *Mach. Learn. Sci. Technol.* **6**, 030602 (2025).

[11] Hong, J., Wang, Z. & Yao, Y. Fault prognosis of battery system based on accurate voltage abnormity prognosis using long short-term memory neural networks. *Appl. Energy* **251**, 113381 (2019).

[12] Hsu, C.-W., Xiong, R., Chen, N.-Y., Li, J. & Tsou, N.-T. Deep neural network battery life and voltage prediction by using data of one cycle only. *Appl. Energy* **306**, 118134 (2022).

[13] Song, Z. et al. Simultaneous identification and control for hybrid energy storage system using model predictive control and active signal injection. *IEEE Trans. Ind. Electron.* **67**, 9768–9778 (2019).

[14] Yue, C. et al. Differential privacy distributed optimization algorithm against adversarial attacks for efficiency optimization of complex industrial processes. *Adv. Eng. Inform.* **62**, 102662 (2024).

[15] Qiu, S. et al. Hard label adversarial attack with high query efficiency against NLP models. *Sci. Rep.* **15**, 9378 (2025). 40102502 10.1038/s41598-025-93566-5 PMC11920284

[16] Sun, H., Sun, J., Zhao, K., Wang, L. & Wang, K. Data-driven ICA-Bi-LSTM-combined lithium battery soh estimation. *Math. Probl. Eng.* **2022**, 1–8 (2022).

[17] Zhang, R. et al. Harmonizing transferability and imperceptibility: A novel ensemble adversarial attack. *IEEE Internet Things J.* **11**(15), 25625–25636 (2024).

[18] Zhou, H. et al. Data reduction for black-box adversarial attacks against deep neural networks based on side-channel attacks. *Comput. Secur.* **153**, 104401 (2025).

[19] Zheng, M., Yan, X., Zhu, Z., Chen, H. & Wu, B. Blackboxbench: A comprehensive benchmark of black-box adversarial attacks. *IEEE Trans. Pattern Anal. Mach. Intell.* **47**, 7867–7885 (2025). 40434861 10.1109/TPAMI.2025.3574432

[20] Ma, T., Zhao, H., Tang, L., Xue, M. & Liu, J. Efficient black-box attack with surrogate models and multiple universal adversarial perturbations. *Sci. Rep.* **15**, 17372 (2025). 40389546 10.1038/s41598-025-87529-z PMC12089264

[21] Khedr, Y. M., Liu, X., Lu, H. & He, K. Transferable adversarial attacks against face recognition using surrogate model fine-tuning. *Appl. Soft Comput.* **174**, 112983 (2025).

[22] Lee, S., Kim, H., Lee, W. & Son, Y. Black-box adversarial examples via frequency distortion against fault diagnosis systems. *Appl. Soft Comput.* **171**, 112828 (2025).

[23] Kim, H., Lee, S., Lee, J., Lee, W. & Son, Y. Evaluating practical adversarial robustness of fault diagnosis systems via spectrogram-aware ensemble method. *Eng. Appl. Artif. Intell.* **130**, 107980 (2024).

[24] Goodfellow, I. J., Shlens, J. & Szegedy, C. Explaining and harnessing adversarial examples. *arXiv preprint* arXiv:1412.6572 (2014).

[25] Madry, A., Makelov, A., Schmidt, L., Tsipras, D. & Vladu, A. Towards deep learning models resistant to adversarial attacks. *arXiv preprint* arXiv:1706.06083 (2017).

[26] Xie, C. *et al.* Improving transferability of adversarial examples with input diversity. In *Proceedings of the IEEE/CVF Conference on Computer Vision and Pattern Recognition*, 2730–2739 (2019).

[27] Yao, L., Fang, Z., Xiao, Y., Hou, J. & Fu, Z. An intelligent fault diagnosis method for lithium battery systems based on grid search support vector machine. *Energy* **214**, 118866 (2021).

[28] Li, B., Shu, J. & Cui, F. Research on series arc fault detection method household loads based on voltage signals. *Sci. Rep.* **15**, 27324 (2025). 40717143 10.1038/s41598-025-12760-7 PMC12301451

[29] Kim, J. *et al.* Discrete wavelet transform-based characteristic analysis and soh diagnosis for a li-ion cell. In *Proceedings of The 7th International Power Electronics and Motion Control Conference*, vol. 3, 2218–2223 (IEEE, 2012).

[30] Tang, T. & Yuan, H. A hybrid approach based on decomposition algorithm and neural network for remaining useful life prediction of lithium-ion battery. *Reliab. Eng. Syst. Safety* **217**, 108082 (2022).

[31] Jiang, J., Li, T., Chang, C., Yang, C. & Liao, L. Fault diagnosis method for lithium-ion batteries in electric vehicles based on isolated forest algorithm. *J. Energy Storage* **50**, 104177 (2022).

[32] Kollmeyer, P., Vidal, C., Naguib, M. & Skells, M. Lg 18650hg2 li-ion battery data and example deep neural network xev soc estimator script. *Mendeley Data* **3**, 2020 (2020).

[33] Vennam, G., Sahoo, A. & Ahmed, S. A survey on lithium-ion battery internal and external degradation modeling and state of health estimation. *J. Energy Storage* **52**, 104720 (2022).

[34] Xiong, R., Li, L. & Tian, J. Towards a smarter battery management system: A critical review on battery state of health monitoring methods. *J. Power Sources* **405**, 18–29 (2018).

[35] Tang, X. et al. Recovering large-scale battery aging dataset with machine learning. *Patterns* **2**, 100302 (2021). 34430924 10.1016/j.patter.2021.100302 PMC8369168

[36] Lin, Y.-H. & Jiao, X.-L. Adaptive kernel auxiliary particle filter method for degradation state estimation. *Reliab. Eng. Syst. Safety* **211**, 107562 (2021).

[37] Gao, Y., Liu, K., Zhu, C., Zhang, X. & Zhang, D. Co-estimation of state-of-charge and state-of-health for lithium-ion batteries using an enhanced electrochemical model. *IEEE Trans. Ind. Electron.* **69**, 2684–2696 (2021).

[38] Han, X. et al. A review on the key issues of the lithium ion battery degradation among the whole life cycle. *ETransportation* **1**, 100005 (2019).

[39] Zhao, H. et al. State of health estimation for lithium-ion batteries based on hybrid attention and deep learning. *Reliab. Eng. Syst. Safety* **232**, 109066 (2023).

[40] Ren, Z. & Du, C. A review of machine learning state-of-charge and state-of-health estimation algorithms for lithium-ion batteries. *Energy Rep.* **9**, 2993–3021 (2023).

[41] Liu, Q. et al. Physics-guided TL-LSTM network for early-stage degradation trajectory prediction of lithium-ion batteries. *J. Energy Storage* **106**, 114736 (2025).

[42] Lu, S. et al. A knowledge distillation-based network compression framework for lifecycle management of lithium-ion batteries: S. Lu et al. *J. Supercomput.* **81**(10), 1147 (2025).

[43] Tian, J., Xiong, R. & Shen, W. A review on state of health estimation for lithium ion batteries in photovoltaic systems. *ETransportation* **2**, 100028 (2019).

[44] Lin, H.-T., Liang, T.-J. & Chen, S.-M. Estimation of battery state of health using probabilistic neural network. *IEEE Trans. Ind. Inf.* **9**, 679–685 (2012).

[45] Dubarry, M., Truchot, C. & Liaw, B. Y. Synthesize battery degradation modes via a diagnostic and prognostic model. *J. Power Sources* **219**, 204–216 (2012).

[46] Fan, F., Xu, Y., Zhang, R. & Wan, T. Whole-lifetime coordinated service strategy for battery energy storage system considering multi-stage battery aging characteristics. *J. Modern Power Syst. Clean Energy* **10**, 689–699 (2021).

[47] Papernot, N. *et al.* Practical black-box attacks against machine learning. In *Proceedings of the 2017 ACM on Asia Conference on Computer and Communications Security*, 506–519 (2017).

[48] Choi, Y., Park, J., Lee, J. & Kim, H. Exploring diverse feature extractions for adversarial audio detection. *IEEE Access* **11**, 2351–2360 (2023).

[49] Zhou, W. *et al.* Transferable adversarial perturbations. In *Proceedings of the European Conference on Computer Vision (ECCV)*, 452–467 (2018).

[50] Dong, Y., Pang, T., Su, H. & Zhu, J. Evading defenses to transferable adversarial examples by translation-invariant attacks. In *Proceedings of the IEEE/CVF Conference on Computer Vision and Pattern Recognition*, 4312–4321 (2019).

[51] Sangiri, J. B., Kulshreshtha, T., Ghosh, S., Maiti, S. & Chakraborty, C. A novel methodology to estimate the state-of-health and remaining-useful-life of a li-ion battery using discrete fourier transformation. *J. Energy Storage* **46**, 103849 (2022).

[52] Chang, C., Wang, Q., Jiang, J., Jiang, Y. & Wu, T. Voltage fault diagnosis of a power battery based on wavelet time-frequency diagram. *Energy* **278**, 127920 (2023).

[53] Kim, J. Discrete wavelet transform-based feature extraction of experimental voltage signal for li-ion cell consistency. *IEEE Trans. Veh. Technol.* **65**, 1150–1161 (2015).

[54] Shen, M. & Huang, R. Backdoor attacks with wavelet embedding: Revealing and enhancing the insights of vulnerabilities in visual object detection models on transformers within digital twin systems. *Adv. Eng. Inform.* **60**, 102355 (2024).

[55] Debnath, L. *Wavelet transforms and time-frequency signal analysis* (Springer Science & Business Media, 2012).

[56] Charmchi, H. & Salehi, J. A. Outer-product matrix representation of optical orthogonal codes. *IEEE Trans. Commun.* **54**, 983–989 (2006).

[57] Sun, Z., Sarma, P., Sethares, W. & Liang, Y. Learning relationships between text, audio, and video via deep canonical correlation for multimodal language analysis. In *Proceedings of the AAAI Conference on Artificial Intelligence*, 8992–8999 (2020).

[58] Veselá, A. et al. Infrared spectroscopy and outer product analysis for quantification of fat, nitrogen, and moisture of cocoa powder. *Anal. Chim. Acta* **601**, 77–86 (2007). 17904472 10.1016/j.aca.2007.08.039

[59] Lin, T.-Y., RoyChowdhury, A. & Maji, S. Bilinear cnn models for fine-grained visual recognition. In *Proceedings of the IEEE international conference on computer vision*, 1449–1457 (2015).

[60] Lee, S., Choi, C. & Son, Y. Relation-preserving masked modeling for semi-supervised time-series classification. *Inf. Sci.* **681**, 121213 (2024).

[61] Ismail Fawaz, H., Forestier, G., Weber, J., Idoumghar, L. & Muller, P.-A. Deep learning for time series classification: a review. *Data Min. Knowl. Discov.* **33**, 917–963 (2019).

[62] Karim, F., Majumdar, S., Darabi, H. & Chen, S. LSTM fully convolutional networks for time series classification. *IEEE Access* **6**, 1662–1669 (2017).

[63] Elsayed, N., Maida, A. S. & Bayoumi, M. Deep gated recurrent and convolutional network hybrid model for univariate time series classification. *arXiv preprint* arXiv:1812.07683 (2018).

[64] Bai, S., Kolter, J. Z. & Koltun, V. An empirical evaluation of generic convolutional and recurrent networks for sequence modeling. *arXiv preprint* arXiv:1803.01271 (2018).

[65] Zhang, W., Peng, G., Li, C., Chen, Y. & Zhang, Z. A new deep learning model for fault diagnosis with good anti-noise and domain adaptation ability on raw vibration signals. *Sensors* **17**, 425 (2017). 28241451 10.3390/s17020425 PMC5336047

[66] Chen, C.-C., Liu, Z., Yang, G., Wu, C.-C. & Ye, Q. An improved fault diagnosis using 1d-convolutional neural network model. *Electronics* **10**, 59 (2020).

[67] Zhou, H. *et al.* Informer: Beyond efficient transformer for long sequence time-series forecasting. In *Proceedings of the AAAI conference on artificial intelligence*, 11106–11115 (2021).

[68] Wang, F. et al. Soh estimation for lithium-ion batteries using the distribution of relaxation time and feature optimized multilayer perceptron. *iScience* **28**, 113443 (2025). 41054534 10.1016/j.isci.2025.113443 PMC12496215

[69] Toughzaoui, Y. et al. State of health estimation and remaining useful life assessment of lithium-ion batteries: A comparative study. *J. Energy Storage* **51**, 104520 (2022).

[70] Cui, S. & Joe, I. A dynamic spatial-temporal attention-based GRU model with healthy features for state-of-health estimation of lithium-ion batteries. *IEEE Access* **9**, 27374–27388 (2021).

[71] Bhattacharjee, A., Verma, A., Mishra, S. & Saha, T. K. Estimating state of charge for xEV batteries using 1d convolutional neural networks and transfer learning. *IEEE Trans. Veh. Technol.* **70**, 3123–3135 (2021).

[72] Cai, L. et al. A multi-fault diagnostic method based on category-reinforced domain adaptation network for series-connected battery packs. *J. Energy Storage* **60**, 106690 (2023).

[73] Bockrath, S., Lorentz, V. & Pruckner, M. State of health estimation of lithium-ion batteries with a temporal convolutional neural network using partial load profiles. *Appl. Energy* **329**, 120307 (2023).

[74] Zhou, R. et al. Remaining useful life prediction of lithium-ion batteries based on informer model with multi-feature extraction and bayesian optimization. *Chem. Eng. Sci.* **316**, 121878 (2025).

[75] Peng, Y., Hou, Y., Song, Y., Pang, J. & Liu, D. Lithium-ion battery prognostics with hybrid gaussian process function regression. *Energies* **11**, 1420 (2018).

[76] Uelwer, T., Michels, F. & De Candido, O. Learning to detect adversarial examples based on class scores. In *German Conference on Artificial Intelligence (Künstliche Intelligenz)*, 233–240 (Springer, 2021).

[77] Wang, X., Li, S., Liu, M., Wang, Y. & Roy-Chowdhury, A. K. Multi-expert adversarial attack detection in person re-identification using context inconsistency. In *Proceedings of the IEEE/CVF International Conference on Computer Vision*, 15097–15107 (2021).

[78] Popov, P. et al. A simple but tough-to-beat baseline for fMRI time-series classification. *Neuroimage* **303**, 120909 (2024). 39515403 10.1016/j.neuroimage.2024.120909 PMC11625415

[79] Teraoka, J. & Tamura, K. Adversarial attacks for time series classification using partial perturbations. *Int. J. Serv. Knowl. Manag.* **8** (2024).

